# New roles for Fc receptors in neurodegeneration-the impact on Immunotherapy for Alzheimer's Disease

**DOI:** 10.3389/fnins.2014.00235

**Published:** 2014-08-21

**Authors:** James P. Fuller, Jeffrey B. Stavenhagen, Jessica L. Teeling

**Affiliations:** ^1^CNS Inflammation Group, Centre for Biological Sciences, University of SouthamptonSouthampton, UK; ^2^Biologics, H. Lundbeck A/SCopenhagen, Denmark

**Keywords:** Fc receptors, Alzheimer's Disease, immunotherapy, neuroinflammation, cytokines, ARIAs, auto-antibodies

## Abstract

There are an estimated 18 million Alzheimer's disease (AD) sufferers worldwide and with no disease modifying treatment currently available, development of new therapies represents an enormous unmet clinical need. AD is characterized by episodic memory loss followed by severe cognitive decline and is associated with many neuropathological changes. AD is characterized by deposits of amyloid beta (Aβ), neurofibrillary tangles, and neuroinflammation. Active immunization or passive immunization against Aβ leads to the clearance of deposits in transgenic mice expressing human Aβ. This clearance is associated with reversal of associated cognitive deficits, but these results have not translated to humans, with both active and passive immunotherapy failing to improve memory loss. One explanation for these observations is that certain anti-Aβ antibodies mediate damage to the cerebral vasculature limiting the top dose and potentially reducing efficacy. Fc gamma receptors (FcγR) are a family of immunoglobulin-like receptors which bind to the Fc portion of IgG, and mediate the response of effector cells to immune complexes. Data from both mouse and human studies suggest that cross-linking FcγR by therapeutic antibodies and the subsequent pro-inflammatory response mediates the vascular side effects seen following immunotherapy. Increasing evidence is emerging that FcγR expression on CNS resident cells, including microglia and neurons, is increased during aging and functionally involved in the pathogenesis of age-related neurodegenerative diseases. Therefore, we propose that increased expression and ligation of FcγR in the CNS, either by endogenous IgG or therapeutic antibodies, has the potential to induce vascular damage and exacerbate neurodegeneration. To produce safe and effective immunotherapies for AD and other neurodegenerative diseases it will be vital to understand the role of FcγR in the healthy and diseased brain. Here we review the literature on FcγR expression, function and proposed roles in multiple age-related neurological diseases. Lessons can be learnt from therapeutic antibodies used for the treatment of cancer where antibodies have been engineered for optimal efficacy.

## Introduction

Alzheimer's disease (AD) is a devastating illness with a hugely detrimental effect on the quality of life of patients and their families. An estimated 18 million people worldwide suffer from AD and with an ever ageing population; the number of cases will increase. The currently approved treatments for AD are: inhibitors of the enzyme acetyl-cholinesterase, and Memantine which is a blocker of the neurotransmitter channel for glutamate (NMDA). Both these therapies are able to transiently improve cognition (Reisberg et al., 2003; Tariot et al., 2004), but no treatment is available that can modify disease progression. The increasing number of individuals suffering from AD and the burden placed on our healthcare services, makes developing effective AD therapeutics an urgent unmet clinical need.

AD patients initially present with episodic memory loss which is followed by severe cognitive decline, but pathological changes begin decades before clinical symptoms arise. The disease is characterized by a number of neuropathological changes including: the deposition of misfolded proteins in the brain as extra-cellular plaques and intracellular tangles, loss of synapses and neurons and an increased number and activation of glial cells. The primary component of plaques was identified as amyloid beta (Aβ), a 39–43 amino acid peptide cleaved from the amyloid precursor protein (APP) (Masters et al., 1985; Allsop et al., 1988). Later it was discovered that mutations in the APP gene (Goate et al., 1991; Mullan et al., 1992), or increased copy number of APP found in Down's syndrome are associated with early onset AD pathology (Olson and Shaw, 1969). These histological and genetic findings formed the basis of the amyloid cascade hypothesis, which states that increased production or accumulation of Aβ underlies the pathology of AD (Hardy and Selkoe, 2002). This hypothesis has driven most AD research in the last 20 years, resulting in the development of therapies targeted to reduce Aβ production or clear Aβ deposits from the brain.

Immunotherapy is one strategy that has been developed to clear Aβ from the brains of AD patients, with a number of vaccines and monoclonal antibodies against Aβ that have been tested in clinical trials. Despite early optimism from successful pre-clinical work, this approach has not translated into disease modifying therapies. In 2012 two phase III clinical trials for anti-Aβ antibodies Bapineuzumab (AAB-001, ELN115727) and Solanezumab (EXPEDITION 1 and EXPEDITION 2) failed to meet primary clinical endpoints of improvement in cognition (Doody et al., 2014; Salloway et al., 2014). A major safety finding was that Bapineuzumab caused vascular side effects designated amyloid related imaging abnormalities (ARIAs). This led to the discontinuation of the 2 mg/kg dose, which may have decreased efficacy (Salloway et al., 2010). In spite of these disappointing clinical results interest in this form of therapy persists with second generation antibodies that are currently being tested in AD prevention trials.

The mechanism underlying the development of side effects is poorly understood, and only a limited number of studies have investigate if the effector function of the therapeutic antibodies is critical for efficacy. Fc gamma receptors (FcγRs) bind to the constant domain of IgG, and are expressed on a wide variety of cell types including CNS macrophages- microglia. Activation of FcγR can result in a pro-inflammatory response including the release of cytokines and other mediators (Carbone et al., 2013). Experimental models of AD and observations from clinical trials have provided evidence that activating FcγRs may be responsible for the activation of microglia following immunotherapy, and the associated side effects (Wilcock et al., 2006; Adolfsson et al., 2012; Freeman et al., 2012). There are a number of inflammatory changes within the CNS during ageing, which are further affected by AD, including increased expression of all FcγRs on microglial cells and/or perivascular macrophages (Peress et al., 1993; Cribbs et al., 2012). This is functionally relevant as increased expression of activating FcγRs on microglia could drive an exacerbated response to therapeutic antibodies, possibly explaining the side effects observed in the Bapineuzumab clinical trials.

This review will describe the emerging roles for specific FcγRs in the underlying pathology of neurodegeneration and the potential for FcγR mediated tissue damage in the CNS. This will be discussed in the context of immunotherapy for AD and the considerations that should be made before the development of next generation antibodies targeting CNS antigens.

## Fc receptors

FcγRs bind to the constant region of IgG and are expressed on the surface of a wide range of immune effector cells. Human FcγRs can be functionally divided into three classes: activating (FcγRI, FcγRIIa, FcγRIIc, and FcγRIIIa), inhibitory (FcγRIIb) or gpi linked decoy (FcγRIIIb). There are a number of subclasses of human IgG (IgG1, IgG2, IgG3, and IgG4), each with varying affinity for the different FcγRs (Bruhns et al., 2009). There are four known murine FcγRs: activating (FcγRI, FcγRIII, and FcγRIV) and inhibitory (FcγRIIb) (Nimmerjahn and Ravetch, 2008). Similar to humans mice have 4 IgG subclasses (IgG1, IgG2a, IgG2b, and IgG3), but it should be noted that the nomenclature is different between species, and therefore human IgG1 is not homologous to murine IgG1, and its effector function is instead more similar to murine IgG2a.

**Figure 1:**
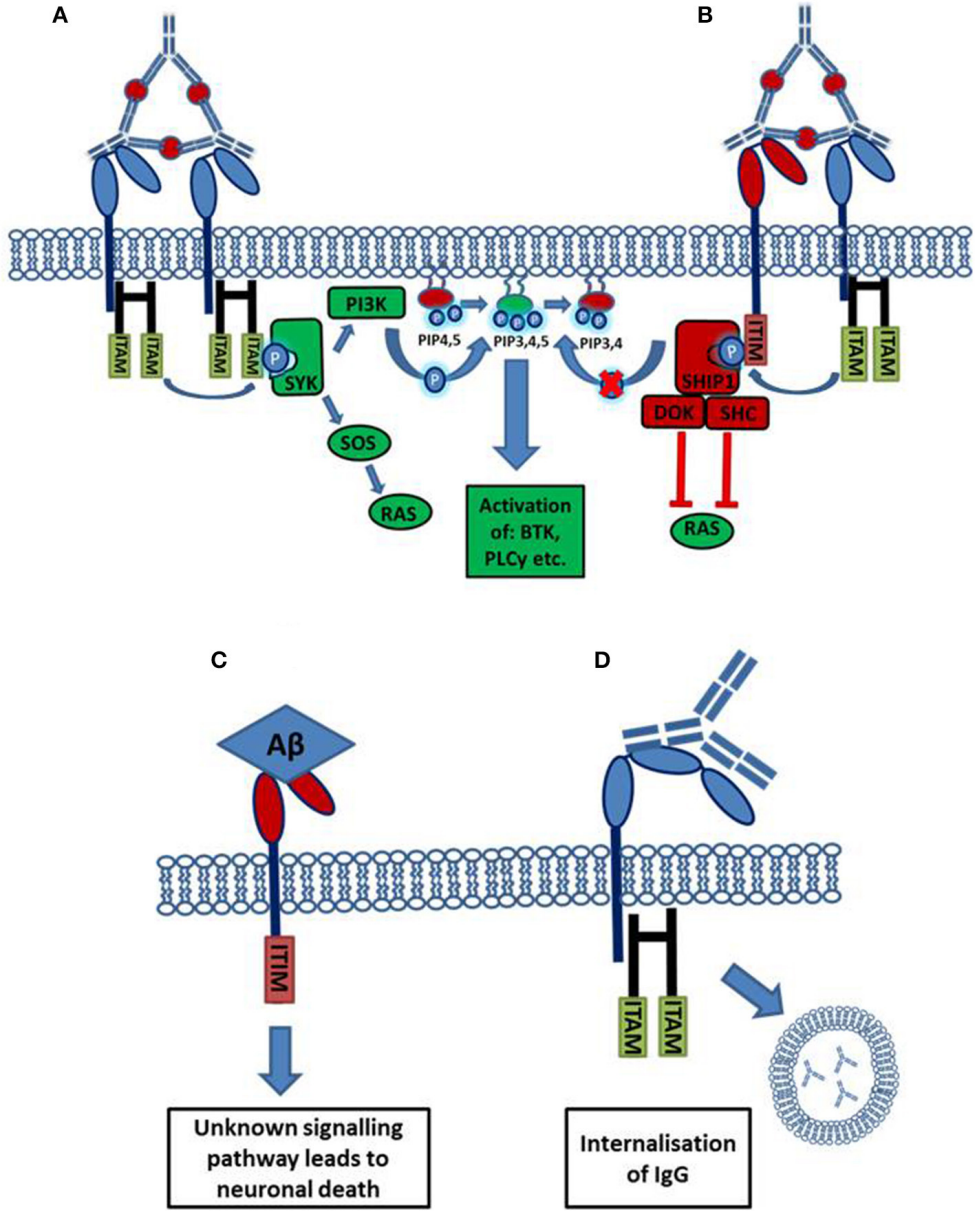
**Activation or inhibition of a cell by Fc receptor ligation of IgG immune complexes. (A)** Cross linking of activating FcγRs by IgG immune complexes results in the phosphorylation of cytoplasmic ITAM motifs. This allows the recruitment of SH2 domain containing kinases of the SYK family. These kinases activate pathways such as the RAS and PI3K pathways resulting in increased cellular calcium and activation of the cell. **(B)** The cross linking of an inhibitory receptor to an activating receptor results in the phosphorylation of an ITIM, leading to the recruitment of the phosphatase SHIP1. SHIP1 removes the 5'phopshate from PiP_345_ inhibiting downstream PI3K signaling, and also interacts with other adaptor proteins to inhibit other pathways. **(C)** Aβ binds with high affinity to the inhibitory FcγRIIb (*K*_D_ = 5.67 × 10^−8^ M). Through an unknown signaling pathway, the ligation of Aβ causes the loss of FcγRIIb expressing neurons. **(D)** FcγRI possesses an extra immunoglobulin like domain compared to other FcγRs. This allows the high affinity binding of monomeric IgG, ligation of monomeric IgG by FcγRI expressing neurons, facilitating antibody uptake.

Activating Fc receptors (with the exception of human FcγRIIa) are associated with and signal through a separate Fc gamma chain (Fcγ chain) which contains immune-tyrosine activation motifs (ITAMs). A common model used to study the roles of activating FcγRs *in vivo* are mice deficient for this Fcγ chain, who lack expression of functional activating FcγRs. Activating FcγR deficient mice show: decreased antibody mediated phagocytosis, abnormal platelet activation and an attenuate immune response to immune complexes (Takai et al., 1994). However, some of these effects may be mediated by other immune receptors, such as c-type lectins, which also depend on Fcγ chain signaling (Geijtenbeek and Gringhuis, 2009). In contrast to activating Fcγ receptors, the expression and therefore function of the inhibitory Fcγ receptor (FcγRIIb) is maintained. Human FcγRIIa carries an intrinsic ITAM in its cytoplasmic domain. Ligation of IgG-immune complexes by activating FcγRs results in the crosslinking of the receptor and the phosphorylation of ITAMs in the cytoplasmic chain. This forms a binding site for the Spleen tyrosine kinase (Syk), which then activates downstream signaling cascades such as the PI3K pathway. Cellular calcium levels are increased and the cell becomes activated which can result in: proliferation, cytokine/chemokine release, phagocytosis and antigen presentation (Nimmerjahn and Ravetch, 2008). The inhibitory FcγRIIb signals through an intrinsic cytoplasmic immuno tyrosine inhibitory motif (ITIM), cross-linking with an activating receptor results in ITIM phosphorylation leading to the inhibition of cellular activation (Nimmerjahn and Ravetch, 2008). The process of FcγR mediated activation or inhibition of an effector cell is outlined in Figure 1.

The functional consequences of FcγR ligation depends on the ratio of activating to inhibitory FcγRs expressed on the effector cell. Cells that have a high ratio of activating to inhibitory FcγRs are more prone to an uncontrolled immune response; this is demonstrated by FcγRIIb^−/−^ mice which have an exacerbated response to autoantibodies inducing more tissue damage (Clynes et al., 1999; Yuasa et al., 1999). The response is also determined by the subclass; for example human IgG1 has higher affinity for activating FcγRs as compared to human IgG4, and will induce a more pro-inflammatory response (Bruhns et al., 2009). Activation of macrophages, including microglia, through FcγRs results in phagocytosis and polarization to an M2b phenotype which has attributes of both M1 and M2 macrophages. M2b macrophages release high levels of pro inflammatory cytokines such as TNFα and IL-1β as well as nitric oxide, all having potent tissue damaging properties (Mosser, 2003; Mantovani et al., 2004; Mosser and Edwards, 2008). In the context of immunotherapy anti-Aβ antibodies reaching the CNS will bind to and coat plaques; this promotes activation of microglia through FcγRs, resulting in the phagocytosis of the antibody-coated plaques and release of pro-inflammatory mediators. It should be noted that studies in FcγR deficient mice and application of antibodies with reduced or no effector function have shown that there are mechanisms of plaque removal independent of FcγR binding (Bacskai et al., 2002; Das et al., 2003; Wilcock et al., 2006).

### Humoral immunity in the CNS

#### FcγR expression on CNS cells

Although macrophages and microglia have been implicated as effector cells in the CNS following immunotherapy, recent data suggest a more broad expression pattern of FcRs in the CNS. Increasing evidence suggest that multiple cell types within the CNS express FcγRs, and changes in expression patterns occur in response to different stimuli. Tables 1, 2 summarize the expression pattern and function of FcγRs on CNS cells in humans and mice respectively, under healthy and diseased conditions.

**Table 1:** **Expression of FcγRs on murine CNS cells**.

**Cell type**	**Fcγ Rs expressed**	**Conditions for expression**	**References**
Microglia	FcγRI, FcγRIIb, FcγRIII, and FcγRIV	All↑: Neurodegeneration ± systemic LPS, immune complex formation in retina FcγRII/III↑: Amyloid beta immunotherapy or arthus reaction in the brain FcγRI↑: ageing, especially in white matter regions	Wilcock et al., 2004b; Lunnon et al., 2011; Hart et al., 2012; Teeling et al., 2012; Murinello et al., 2014
Neurons	FcγRIIb, FcγRIV FcγRII/III	FcγRIIb↑: Aβ treatment FcγRIV↑: APOE^-/-^ genetic background	Kam et al., 2013 Fernandez-Vizarra et al., 2012
Astrocytes	FcγRI	FcγRI↑: increased CNS IgG	Li et al., 2008
Oligo-dendrocytes	Fcγ chain	Fcγ chain is expressed by oligodendrocyte precursor cells	Nakahara et al., 2003
Endothelial cells	FcRn	FcRn constitutively expressed on CNS endothelium	Deane et al., 2005

*This table shows the expression patterns of FcγRs on different murine CNS cell types. The conditions in which up or down regulation of specific FcγRs has been observed have been recorded*.

**Table 2:** **Expression of FcγRs on human CNS cells**.

**Cell type**	**FcRs expressed**	**Conditions for expression**	**References**
Microglia	FcγRI, FcγRIIa, FcγRIIb, FcγRIIIa	All↑ Alzheimer's disease, multiple sclerosis FcγRI↑ Parkinson's disease FcγRI and FcγRIIb↑ Alzheimer's disease after plaque clearance FcγRIIa and FcγRIIb↑ Age related macular degeneration	Peress et al., 1993; Ulvestad et al., 1994; Orr et al., 2005; Zotova et al., 2013; Murinello et al., 2014
Neurons	FcγRI, FcγRIIb	FcRI Expressed constitutively on sensory and motor neurons FcRIIb↑ Alzheimer's disease	Mohamed et al., 2002; Andoh and Kuraishi, 2004 Kam et al., 2013

*This table shows the expression patterns of FcγRs on different human CNS cell types. The conditions in which up or down regulation of specific FcγRs has been observed have been recorded. There are a limited number of studies examining FcγR expression for most human CNS cell types*.

Human microglia express: FcγRI, FcγRIIa, FcγRIIb, and FcγRIIIa albeit at very low levels under normal conditions. The expression is increased on microglia in the CNS of patients with MS (Ulvestad et al., 1994), and a number of neurodegenerative conditions. In AD ramified microglia in the parenchyma, but especially those associated with plaques express higher levels of: FcγRI, FcγRII, and FcγRIII compared to age matched controls (Peress et al., 1993). Interestingly the expression of FcγRI and FcγRIIb are decreased on microglia of AD patients whose plaques were cleared by active immunotherapy (Zotova et al., 2013). Age related macular degeneration is associated with an increased number of CD45+ leukocytes expressing activating FcγRIIa (and to a lesser extent inhibitory FcγRIIb) at the choroid-retinal epithelial cell interface (Murinello et al., 2014). Finally increased FcγRI microglial expression is found in the CNS of patients with Parkinson's Disease (Orr et al., 2005). Murine microglia express all known FcγRs: FcγRI, FcγRIIb, FcγRIII, and FcγRIV. The expression of these receptors is increased in response to a number of different insults to the CNS. All four FcγRs are up regulated on microglia in response to experimental induced neurodegeneration (prion disease) and further upregulated by systemic inflammation, whilst up-regulation of FcγRII/III has been observed in TG2576 APP mice (Wilcock et al., 2004b; Lunnon et al., 2011). We showed that type III hypersensitivity (or Arthus reaction) in the CNS (brain and retina) induces robust expression of: FcγRI, FcγRIIb, FcγRIII, and FcγRIV on microglia followed by a robust neuro-inflammatory response (Teeling et al., 2012; Murinello et al., 2014). Normal ageing is also associated with increased FcγR immuno-reactivity, and microglia especially those in white matter regions of the CNS, show elevated expression of FcγRI (Hart et al., 2012).

The expression of FcγRs on neurons was once controversial, however a number of studies have now documented their expression on neurons both *in vitro* and *in vivo*. Human sensory and motor neurons express the high affinity FcγRI which enable the cells to take up IgG from the surroundings (Mohamed et al., 2002; Andoh and Kuraishi, 2004). The inhibitory FcγRIIb has been detected on healthy neurons in the hippocampus of both mice and humans. The expression of FcγRIIb is increased on neurons in the AD brain, and also in response to treatment with Aβ (Nakamura et al., 2007; Kam et al., 2013). Murine neurons have been found to express FcγRII/III which mediate the uptake of therapeutic anti tau antibodies into neurons (Congdon et al., 2013). Finally neurons in the hippocampus of APOE^−/−^ mice express FcγRIV and signal in response to the higher levels of IgG in the CNS (Fernandez-Vizarra et al., 2012).

There are a limited number of studies investigating FcγR expression on other CNS cell types. FcγRI has been detected on astrocytes cultured *in vitro*, and also on rat astrocytes *in vivo* in response to BBB permeability changes (Li et al., 2008). There is also evidence that the Fcγ chain is required for differentiation of oligodendrocytes, however with other immunoreceptors that also signal through the Fcγ chain it is not possible to conclude that FcγRs are required (Nakahara et al., 2003). Finally, murine CNS endothelial cells express the neonatal Fc receptor (FcRn). FcRn has been found to mediate the transport of therapeutic anti-Aβ antibodies from the CNS into the periphery (Deane et al., 2005).

#### Immunoglobulin G entry into the CNS

Despite tight control by the BBB, it is apparent that small amounts of IgG enter the healthy brain and it has been estimated that 0.1% of circulating IgG enters the CNS via passive diffusion (Poduslo et al., 1994). However, this may be altered during ageing and/or disease, and associated changes in BBB integrity and interaction with FcRn. For example, under healthy conditions, IgG is removed from the CNS by an efficient process of reverse transcytosis across the BBB (Zhang and Pardridge, 2001), mediated by the neonatal transport receptor, FcRn (Schlachetzki et al., 2002; Deane et al., 2005). This transport of IgG is saturated at high levels of IgG, reducing the rate of IgG efflux. It is widely recognized that a functional blood-brain-barrier (BBB) limits passage of macromolecules and cells from the periphery and that disruption of the BBB by insults such as, ageing, stress and systemic inflammation, obesity is associated with an influx of serum proteins, including IgG (Lu et al., 2001; Diamond et al., 2006). Under these conditions the net influx of IgG would be increased, resulting in accumulation of IgG in the parenchyma, and around cerebral vessels.

### New roles for FcγRs in neurodegeneration

There are emerging roles for different FcγRs in the underlying pathology of neurodegenerative disorders. As outlined in the previous section, increased expression of all FcγRs is consistently reported in human brain tissue of neuro-inflammatory and degenerative diseases including: Parkinson's (PD), AD, and Multiple sclerosis (Nyland et al., 1984; Peress et al., 1993; Ulvestad et al., 1994; Orr et al., 2005; Cribbs et al., 2012). There is evidence that ligation of specific FcγRs in the CNS by IgG and alternate ligands can promote neuroinflammation and/or enhance neurodegeneration.

#### Auto antibodies

Apart from elevated FcγR, increased levels of total IgG in the CNS has also been reported in various neurodegenerative diseases, possibly as a result of an age-related increase in BBB permeability (Bouras et al., 2005). Serum from AD and PD patients are known to contain auto-antibodies to glutamatergic and dopaminergic neurons, which are selectively affected in AD and PD patients respectively. Furthermore, neurons of the substantia nigra (SN) from patients with Idiopathic cases of PD have been found to be immuno-reactive for IgG (Orr et al., 2005). IgG isolated from the sera of PD patients, injected into the brains of mice, specifically binds neurons in the SN. This binding induces neuroinflammation as measured by increased expression of CD11b, and loss of SN neurons (He et al., 2002). The use of Fcγ chain ^−/−^ mice, which lack all activating FcγRs or F(ab')_2_ fragments of PD IgG prevented these responses (He et al., 2002), demonstrating that an FcγR mediated mechanism could drive neurodegeneration in PD. A similar role for FcγRs in the pathology of AD is described. Increased expression of FcγRs (FcγRI, FcγRII, and FcγRIIIa) in disease affected areas of AD patients has been observed on both glial cells and neurons (Peress et al., 1993; Bouras et al., 2005). Application of serum-derived IgG from AD patients, containing neuron specific antibodies, into the forebrain of rats results in the selective reduction of cholinergic neurons, supporting the concept that in AD auto-reactive antibodies could in part drive neuronal loss (Engelhardt et al., 2000). The histological examination of brain tissue from AD patients provides further evidence for a detrimental role of these antibodies, as cholinergic neurons that stain positive for IgG also express markers of degeneration such as caspase 3 (D'Andrea, 2003).

#### Neuronal FcγRs

Evidence for a detrimental role of increased influx of serum derived IgG into the CNS was recently shown in ApoE deficient mice. This model is of interest to AD researchers because the ApoE4 allele is the largest genetic risk factor for sporadic AD (Strittmatter and Roses, 1995). ApoE deficient mice develop many neuropathological changes in common with AD including: increased blood brain barrier permeability, accumulation of intra neuronal Aβ, hyper phosphorylation of Tau and cognitive impairment (Fernandez-Vizarra et al., 2012). A critical role for FcyR was elegantly shown in this experimental model. When crossed onto Fcγ chain deficient mice the double knock out animals have similar increased BBB permeability. However, they are protected from other neuropathological changes including: microgliosis, neuronal damage and cognitive impairment. Fcγ chain deficient mice also do not express other immune receptors such as mincle or dectin 2(Geijtenbeek and Gringhuis, 2009), so this should be taken into account when analyzing this data. However specific knock-down of FcγRIV with siRNA prevents similar effects of IgG on primary neurons *in vitro*. These results imply that it is the interaction between IgG in the brain and FcγR expressing neurons that drive AD-like pathology in these mice. Neurons of AD patients and APP transgenic mice also express the inhibitory receptor FcγRIIb (Kam et al., 2013). FcγRIIb has a low affinity for monomeric IgG1 (*K*_D_ = 9.43 × 10^−6^ M), but binds with high affinity to Aβ (*K*_D_ = 5.67 × 10^−8^ M). Aβ is a potent inducer of neuronal apoptosis, and this effect is ameliorated in FcγRIIb deficient neurons (Kam et al., 2013). It is not currently known if Aβ signals through FcγRIIb using the same signaling pathway as immune complexes. It will be important to understand if cross-linking of neuronal FcγRIIb by immune complexes can induce the same effect.

#### Protection from neurodegeneration in Fcγ chain ^−/−^ mice

Our own studies have provided evidence that the Fcγ chain also contributes to IgG-mediated inflammation and neuronal function. Formation of IgG immune complexes in the mouse brain (Teeling et al., 2012) or the mouse and human retina results in a transient, but robust neuroinflammatory response, that depends on activating FcγRs (Murinello et al., 2014). Further, using an experimental model of neurodegeneration, we show that FcγRs are expressed on microglia and further up-regulated following systemic inflammation. The latter is associated with increased production of pro-inflammatory cytokines, which is attenuated in Fcγ chain deficient mice (Lunnon et al., 2011). The Fcγ chain deficient background is also neuroprotective in other experimental models of neurodegenerative disease, including: ischemic stroke (Komine-Kobayashi et al., 2004) and synuclein-induced neurodegeneration following AAV transfer (Cao et al., 2010, 2012). These results indicate that the Fcγ chain is involved in neurodegeneration, however due to the loss of other immune receptors which signal through the Fcγ chain it is currently not possible to conclude that only FcγRs are involved and further research using models for to test the role for specific FcγR is required.

#### The effects of soluble inflammatory mediators

The ligation of therapeutic antibody coated plaques or auto-antibody coated neurons by activating FcγRs may lead to the polarization of macrophages to a M2b phenotype and the production of a number of cytokines, chemokines, and inflammatory mediators. Receptors for a number of cytokines are expressed on CNS resident cells, so what is the effect of increasing the levels of these mediators on neurons?

Neuronal function is tightly regulated and a small change in homeostasis can be detrimental depending on the levels of cytokines produced. At physiological levels TNFα has important roles in regulating normal brain activity including regulation of synaptic scaling (Stellwagen and Malenka, 2006). Low levels of monomeric IgG in the brain (0.2-20 ug/ml) are observed under healthy conditions and induce low levels of TNFα via tonic signaling of FcγRI expressed on microglia. Under these condition TNFα is neuroprotective against excitotoxicity, but increasing levels of monomeric IgG abrogates this effect eventually becoming neurotoxic (Hulse et al., 2008). At higher levels TNFα, at least *in vitro*, is neurotoxic causing loss of cells by signaling through TNFR1 (Yang et al., 2002). Therefore increased TNFα production as a result of excessive FcγR ligation could be twofold: high levels could induce apoptosis of susceptible neurons or it could interfere with TNFα's regulation of synaptic plasticity. IL-1β is also important for the function of neurons under physiological conditions. Normal concentrations of IL-1β are essential for hippocampal long term potentiation (LTP), however higher pathological levels (>10 ng/ml) results in the inhibition of LTP (Katsuki et al., 1990; Bellinger et al., 1993; Ross et al., 2003). *In vivo* studies are sparse, but *in vitro* studies have provided further evidence of a critical role of FcγR in neuronal damage microglia co-cultured with dopaminergic neurons coated with IgG from the sera of PD patients, results in increased levels of pro-inflammatory cytokines (TNFα), reactive oxygen species synthesis, and the initiation of cell death in co-cultured neurons. The damage to neurons was shown to be dependent on NO production, as iNOS inhibitors but not cytokine blocking antibodies could inhibit neuronal death. This effect was shown to be Fcγ chain-dependent as microglia from Fcγ chain deficient mice fail to induce cytokine/ROS production or neuronal damage (Le et al., 2001). Recently it was shown that microglial activation in response to LPS or Aβ could epigenetically regulate the expression of synaptic proteins, silencing neuroligin gene expression (Bie et al., 2014). The mediators produced by microglia which cause these effects are yet unknown, but it is possible that antibody mediated inflammation could also cause silencing neuroligin gene expression resulting in synapse loss. Therefore the increased production of: TNFα, IL-1β, NO or other inflammatory mediators by M2b polarized microglia could lead to impairment of neuronal function or exacerbation of neuronal/synapse loss in AD patients after treatment.

**Figure 2:**
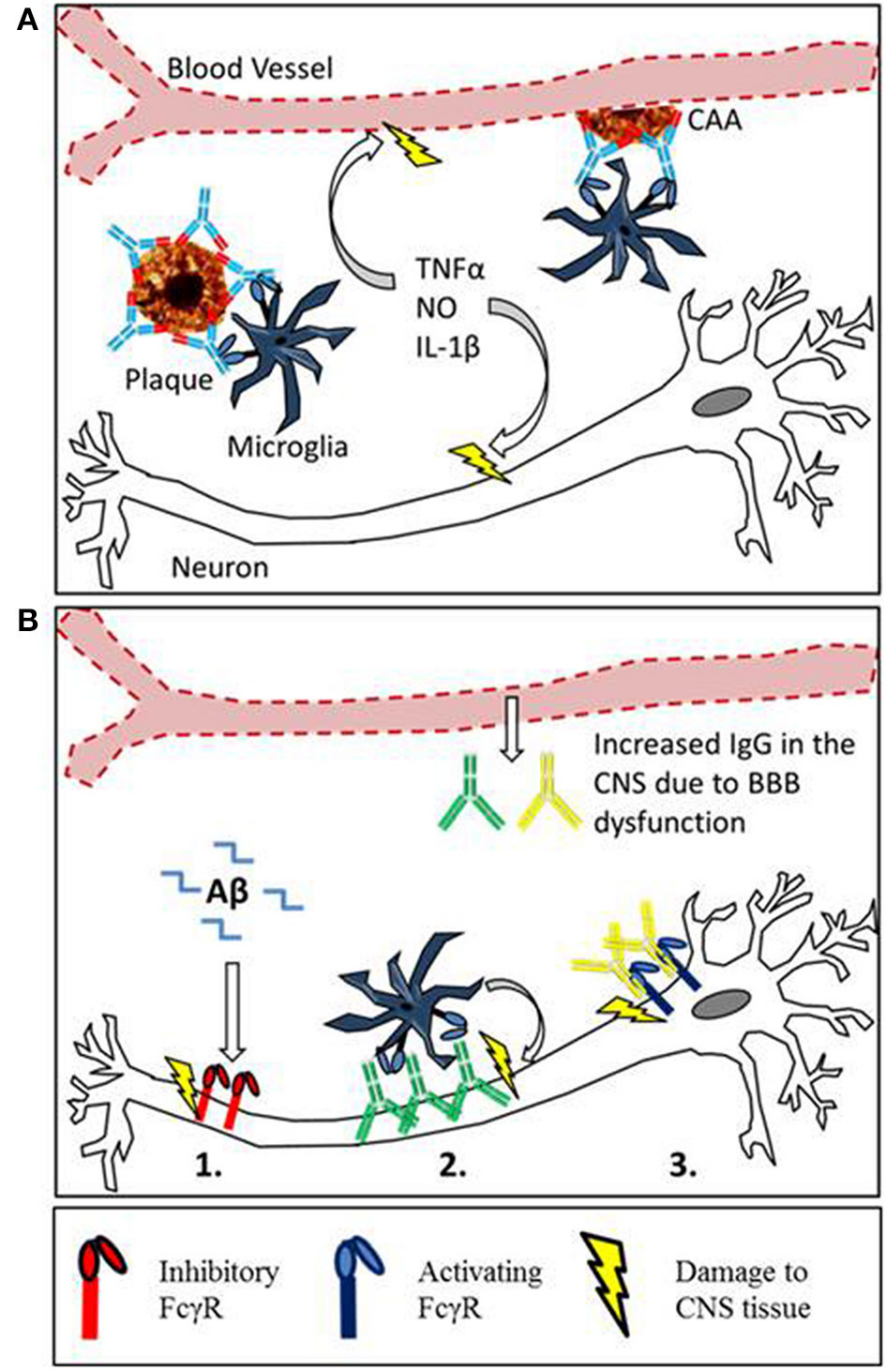
**Mechanisms of Fc receptor mediated tissue damage in the CNS. (A)** The proposed mechanism for inflammatory tissue damage to CNS vasculature and neurons after Anti-Aβ immunotherapy. Therapeutic antibodies penetrate the CNS and bind to deposits of Aβ in the parenchyma and around the blood vessels. Microglia express activating FcγR, and the antibody-Aβ immune complexes cause cross linking and FcγR activation. This results in a localized inflammatory response causing the vascular side effects observed in mice and humans. Furthermore soluble inflammatory mediators produced by this reaction may interfere with neuronal function or induce damage. **(B)** There is an emerging role for Fcγ receptors in neurodegeneration, with 3 different proposed mechanisms of Fc receptor mediated damage to neurons. (1) Inhibitory Fcγ receptor (FcγRIIb) expression has been detected on neurons, and FcRIIb binds Aβ with high affinity. The ligation of Aβ by neuronal FcRIIb results in neuronal death. (2) Autoantibodies against neurons are present in the sera of AD patients and also observed binding to neurons. This could lead to FcγR dependant neuronal loss through antibody dependent cellular cytotoxicity, caused by the ligation of activating Fcγ receptors on microglia. (3) Activating FcγR expression has been detected in certain models with neurodegenerative disease. Ligation of IgG by neuronal Fc receptors in mice results in neuronal loss.

We propose that FcγRs may contribute to the pathology of neurodegenerative diseases through a number of mechanisms (summarized in Figure 2B). It is important to consider this when developing immunotherapies targeting CNS antigens. Targeting Aβ leads to changes in FcγR expression on CNS effector cells, if this alters the ratio of activating to inhibitory FcγR it could exacerbate existing pathology. Furthermore immune complexes formed in the CNS may interact with FcγR expressing cells, including neurons. At the moment the function of FcγR on neurons is unclear but it seems that their ligation can induce neuron loss. It will be important to understand the function and consequence of neuronal FcγR activation in the development of new immunotherapies.

### The importance of FcγR in developing future immunotherapies for alzheimer's disease

#### The role of FcγRs in the clearance of Aβ and associated side effects

The development of anti Aβ immunotherapy has already been reviewed elsewhere (Schenk, 2002; Brody and Holtzman, 2008; Nitsch and Hock, 2008; Morgan, 2009; Lemere and Masliah, 2010), this section will instead focus on the side effects associated with immunotherapy and the putative role of FcγRs in plaque clearance. Passive immunization with anti-Aβ antibodies reduces amyloid deposition and reverses cognitive deficiencies in transgenic APP mice (Bard et al., 2000; DeMattos et al., 2001; Wilcock et al., 2004b). However the reduction in parenchymal amyloid is associated with increased deposition of Aβ around the cerebral vasculature, and an increased incidence of cerebral micro-hemorrhage (Wilcock et al., 2004c). Data from clinical trials has shown that certain antibodies such as Bapineuzumab (hIgG1 anti Aβ) are able to reduce or at least stabilize amyloid load in the brains of AD patients, measured by PET scan (Rinne et al., 2010; Salloway et al., 2014). However MRI scans of Bapineuzumab treated patients have also revealed similar vascular side effects to APP mice. Cerebral Micro-hemorrhage (ARIA-H) was observed at higher frequency in Bapineuzumab treated patients than placebo, whilst vasogenic edema (ARIA-E) was detected in 9.7% of patients treated with bapineuzumab and 0% in the placebo group (Salloway et al., 2010). Due to these side effects experienced by the patients, the top dose of bapineuzumab given in phase III was abandoned, potentially contributing to the lack of efficacy (Salloway et al., 2010). One mechanism for the clearance of plaques by anti Aβ immunotherapy is through FcγR mediated phagocytosis of plaques by microglia; the involvement of microglia in clearance of Aβ has been reviewed (Morgan, 2009). This phagocytic clearance seems to be a double edged sword, as the clearance of Aβ is accompanied micro-hemorrhage and activation of microglia (Wilcock et al., 2004c). This activation takes the form of increased expression of markers such as CD45 and MHCII, and increased transcript levels of pro inflammatory cytokines and iNOS (Wilcock et al., 2004a,b, 2011). The proposed mechanism for microglial mediated damage to the vasculature and neurons is depicted in Figure 2A. Both microglial activation and micro-hemorrhage are prevented when anti-Aβ antibodies were deglycosylated or produced aglycosal reducing affinity for all FcγRs (Wilcock et al., 2006; Freeman et al., 2012). These findings have been translated into the development of Crenezumab where the selection of an antibody with lower affinity for all FcγRs has alleviated vascular side effects in humans (Adolfsson et al., 2012).

#### Are “primed” AD brains predisposed to an exacerbated response to immunotherapy?

Neuro-inflammation in AD is characterized by an upregulation of immune regulatory receptors expressed on: microglia, astrocytes and perivascular cells as well as increased cytokine concentration in the brain (Peress et al., 1993; Wyss-Coray et al., 2001). A causal role of inflammation in disease is supported by the recent genome wide association studies identifying a number of polymorphisms in immune receptors linked to higher risk of late onset AD in genes such as: Complement receptor 1 (Lambert et al., 2009), TREM2 (Guerreiro et al., 2013), HLA DR (Lambert et al., 2013) and CD33 (Hollingworth et al., 2011). We and others have shown that microglia, and perhaps vascular endothelial cells, are functionally altered adopting a “primed” phenotype with reduced threshold for innate immune activation (Lunnon et al., 2011; Puentener et al., 2012). Stimuli such as systemic bacterial infections can switch these cells into an aggressive phenotype, which may explain the exaggerated cognitive decline in AD patients with low grade systemic inflammation (Holmes et al., 2011). We also showed that the pro-inflammatory response is reduced in Fcγ chain deficient mice suggesting that these receptors or their signaling pathways play a role in microglial priming. FcγR expression in the healthy CNS is low, but altered expression of both activating and inhibitory FcγRs as a result of ageing and/or neuroinflammation has been observed in both animal models (Lunnon et al., 2011; Hart et al., 2012; Teeling et al., 2012) and humans (Peress et al., 1993; Bouras et al., 2005; Cribbs et al., 2012). As the balance of activating and inhibitory FcγRs determines the effector function of the cell, higher expression of immune activating tyrosine motif (ITAM) bearing receptors in the CNS may exacerbate the response following ligation of therapeutic antibodies coating plaques. Neurons use a number of mechanisms to keep inflammatory response in the brain tightly regulated, preventing damage to the CNS- these mechanisms, include CD200 and fractalkine which have been reviewed previously (Hoarau et al., 2011). In AD it has been shown that these pathways are deregulated, and the inhibitory signal is reduced. These observations may have important implications for immunotherapy as primed microglia and/or endothelial cells may differentially respond to therapeutic antibodies. The loss of an inhibitory signal coupled with increased activating FcγR expression could drive an exacerbated response to plaques coated in antibodies. This could, at least partly, explain why patients receiving antibodies have experienced vascular damage. Furthermore this may lead to change in neural function as a result of increased production of pro-inflammatory mediators such as the cytokines IL-1β, TNFα and nitric oxide (NO) (Le et al., 2001; Wilcock et al., 2011).

## Conclusions

The clinical trials to date have shown that immunotherapy against Aβ is able to clear plaques from the brains of AD patients. However they have also highlighted the danger of immune activation within the CNS, which can result in damage to brain tissue. It appears that ligation of activating FcγRs by therapeutic antibodies causes damage to the vasculature of the CNS; we propose that immunotherapy could also impair the function of neurons through mechanisms outlined in Figure 2. We believe that we can learn lessons from cancer immunotherapy where the FcγR affinity of monoclonal antibodies has been optimized by point mutations to improve efficacy. To a certain extent this has been started by AC Immune/Genentech whose new antibody Crenezumab (hIgG4) has lower FcγR affinity than previous antibodies. It may be possible to further improve by optimizing the relative affinity for specific FcγRs, allowing higher levels of plaque clearance with fewer side effects. It is clear that different FcγRs play a role in neurodegeneration by a number of mechanisms; however their normal function in the brain is not well understood. To allow the production of safe and effective CNS immunotherapies it is essential to understand the consequences of antibody accumulation and activation of FcγRs in the CNS. This would allow the selection the most appropriate antibody isotypes or mutants minimizing the risk of adverse events.

### Conflict of interest statement

Jeff B. Stavenhagne is a full time employee of H lundbeck A/S. Jessica L. Teeling and James P. Fuller have received funding from H Lundbeck A/S

## References

[B1] Adolfsson O. Pihlgren M. Toni N. Varisco Y. Buccarello A. L. Antoniello K. (2012). An effector-reduced anti-β-amyloid (Aβ) antibody with unique aβ binding properties promotes neuroprotection and glial engulfment of Aβ. J. Neurosci. 32, 9677–9689 10.1523/JNEUROSCI.4742-11.2012 22787053 PMC6622286

[B2] Allsop D. Wong C. W. Ikeda S. Landon M. Kidd M. Glenner G. G. (1988). Evidence for the origin of cerebral amyloid in Alzheimer's disease drom A Beta protein precursor. Neuropathol. Appl. Neurobiol. 14, 254–255

[B3] Andoh T. Kuraishi Y. (2004). Primary sensory neurons express the high affinity IgG Fc gamma RI receptor and responds to IgG-antigen complex. J. Pharmacol. Sci. 94, 74P 10.1096/fj.02-1169fje

[B4] Bacskai B. J. Kajdasz S. T. McLellan M. E. Games D. Seubert P. Schenk D. (2002). Non-Fc-mediated mechanisms are involved in clearance of amyloid-beta *in vivo* by immunotherapy. J. Neurosci. 22, 7873–7878 12223540 10.1523/JNEUROSCI.22-18-07873.2002 PMC6758112

[B5] Bard F. Cannon C. Barbour R. Burke R. L. Games D. Grajeda H. (2000). Peripherally administered antibodies against amyloid beta-peptide enter the central nervous system and reduce pathology in a mouse model of Alzheimer disease. Nat. Med. 6, 916–919 10.1038/78682 10932230

[B6] Bellinger F. P. Madamba S. Siggins G. R. (1993). Interleukin 1 Beta inhibits synaptic strength and lon-term potentiation in the rat CA1 hippocampus. Brain Res. 628, 227–234 10.1016/0006-8993(93)90959-Q 8313151

[B7] Bie B. Jiang W. Yang H. Xu J. J. Brown D. L. Naguib M. (2014). Epigentic suppresion of neuroligin 1 underlies amyloid induced memory deficiency. Nat. Neurosci. 17, 223–231 10.1038/nn.3618 24441681

[B8] Bouras C. Riederer B. M. Kovari E. Hof P. R. Giannakopoulos P. (2005). Humoral immunity in brain aging and Alzheimer's disease. Brain Res. Rev. 48, 477–487 10.1016/j.brainresrev.2004.09.009 15914253

[B9] Brody D. L. Holtzman D. M. (2008). Active and passive immunotherapy for Neurodegenerative disorders. Annu. Rev. Neurosci. 31, 175–193 10.1146/annurev.neuro.31.060407.125529 18352830 PMC2561172

[B10] Bruhns P. Iannascoli B. England P. Mancardi D. A. Fernandez N. Jorieux S. (2009). Specificity and affinity of human Fc gamma receptors and their polymorphic variants for human IgG subclasses. Blood 113, 3716–3725 10.1182/blood-2008-09-179754 19018092

[B11] Cao S. Standaert D. G. Harms A. S. (2012). The gamma chain subunit of Fc receptors is required for alpha-synuclein-induced pro-inflammatory signaling in microglia. J. Neuroinflammation 9:259 10.1186/1742-2094-9-259 23186369 PMC3526448

[B12] Cao S. Theodore S. Standaert D. G. (2010). Fc gamma receptors are required for NF-kappa B signaling, microglial activation and dopaminergic neurodegeneration in an AAV-synuclein mouse model of Parkinson's disease. Mol. Neurodegener. 5:42 10.1186/1750-1326-5-42 20977765 PMC2975641

[B13] Carbone F. Nencioni A. Mach F. Vuilleumier N. Montecucco F. (2013). Evidence on the pathogenic role of auto-antibodies in acute cardiovascular diseases. Thromb. Haemost. 109, 854–868 10.1160/TH12-10-0768 23446994

[B14] Clynes R. Maizes J. S. Guinamard R. Ono M. Takai T. Ravetch J. V. (1999). Modulation of immune complex-induced inflammation *in vivo* by the coordinate expression of activation and inhibitory Fc receptors. J. Exp. Med. 189, 179–185 10.1084/jem.189.1.179 9874574 PMC1887693

[B15] Congdon E. E. Gu J. P. Sait H. B. R. Sigurdsson E. M. (2013). Antibody uptake into neurons occurs primarily via clathrin-dependent fc gamma receptor endocytosis and is a prerequisite for acute tau protein clearance. J. Biol. Chem. 288, 35452–35465 10.1074/jbc.M113.491001 24163366 PMC3853292

[B16] Cribbs D. H. Berchtold N. C. Perreau V. Coleman P. D. Rogers J. Tenner A. J. (2012). Extensive innate immune gene activation accompanies brain aging, increasing vulnerability to cognitive decline and neurodegeneration: a microarray study. J. Neuroinflammation 9:179 10.1186/1742-2094-9-179 22824372 PMC3419089

[B17] D'Andrea M. R. (2003). Evidence linking neuronal cell death to autoimmunity in Alzheimer's disease. Brain Res. 982, 19–30 10.1016/S0006-8993(03)02881-6 12915236

[B18] Das P. Howard V. Loosbrock N. Dickson D. Murphy M. P. Golde T. E. (2003). Amyloid-beta immunization effectively reduces amyloid deposition in FcR gamma(-/-) knock-out mice. J. Neurosci. 23, 8532–8538 13679422 10.1523/JNEUROSCI.23-24-08532.2003 PMC6740360

[B19] Deane R. Sagare A. Hamm K. Parisi M. LaRue B. Guo H. (2005). IgG-assisted age-dependent clearance of Alzheimer's amyloid beta peptide by the blood-brain barrier neonatal Fc receptor. J. Neurosci. 25, 11495–11503 10.1523/JNEUROSCI.3697-05.2005 16354907 PMC6726020

[B20] DeMattos R. B. Bales K. R. Cummins D. J. Dodart J. C. Paul S. M. Holtzman D. M. (2001). Peripheral anti-A beta antibody alters CNS and plasma A beta clearance and decreases brain A beta burden in a mouse model of Alzheimer's disease. Proc. Natl. Acad. Sci. U.S.A. 98, 8850–8855 10.1073/pnas.151261398 11438712 PMC37524

[B21] Diamond B. Kowal C. Huerta P. T. Aranow C. Mackay M. DeGiorgio L. A. (2006). Immunity and acquired alterations in cognition and emotion: lessons from SLE, in Advances in Immunology, Vol. 89, ed Alt F. W. (New York, NY: Academic Press Inc), 289–320 10.1016/S0065-2776(05)89007-8 16682277

[B22] Doody R. S. Thomas R. G. Farlow M. Iwatsubo T. Vellas B. Joffe S. (2014). Phase 3 trials of solanezumab for mild-to-moderate Alzheimer's disease. N. Engl. J. Med. 370, 311–321 10.1056/NEJMoa1312889 24450890

[B23] Engelhardt J. I. Le W. D. Siklos L. Obal I. Boda K. Appel S. H. (2000). Stereotaxic injection of IgG from patients with Alzheimer disease initiates injury of cholinergic neurons of the basal forebrain. Arch. Neurol. 57, 681–686 10.1001/archneur.57.5.681 10815134

[B24] Fernandez-Vizarra P. Lopez-Franco O. Mallavia B. Higuera-Matas A. Lopez-Parra V. Ortiz-Munoz G. (2012). Immunoglobulin G Fc receptor deficiency prevents Alzheimer-like pathology and cognitive impairment in mice. Brain 135, 2826–2837 10.1093/brain/aws195 22961553

[B25] Freeman G. B. Brown T. P. Wallace K. Bales K. R. (2012). Chronic administration of an aglycosylated murine antibody of ponezumab does not worsen microhemorrhages in aged Tg2576 mice. Curr. Alzheimer Res. 9, 1059–1068 10.2174/156720512803569064 22631613

[B26] Geijtenbeek T. B. H. Gringhuis S. I. (2009). Signalling through C-type lectin receptors: shaping immune responses. Nat. Rev. Immunol. 9, 465–479 10.1038/nri2569 19521399 PMC7097056

[B27] Goate A. Chartier-Harlin M.-C. Mullan M. Brown J. Crawford F. Fidani L. (1991). Segregation of a missense mutation in the amyloid precursor protein gene with familial Alzheimer's disease. Nature 349, 704–706 10.1038/349704a0 1671712

[B28] Guerreiro R. Wojtas A. Bras J. Carrasquillo M. Rogaeva E. Majounie E. (2013). TREM2 variants in Alzheimer's disease. N. Engl. J. Med. 368, 117–127 10.1056/NEJMoa1211851 23150934 PMC3631573

[B29] Hardy J. Selkoe D. J. (2002). Medicine - The amyloid hypothesis of Alzheimer's disease: progress and problems on the road to therapeutics. Science 297, 353–356 10.1126/science.1072994 12130773

[B30] Hart A. D. Wyttenbach A. Perry V. H. Teeling J. L. (2012). Age related changes in microglial phenotype vary between CNS regions: grey versus white matter differences. Brain Behav. Immun. 26, 754–765 10.1016/j.bbi.2011.11.006 22155499 PMC3381227

[B31] He Y. Le W.-D. Appel S. H. (2002). Role of Fcγ receptors in nigral cell injury induced by Parkinson disease immunoglobulin injection into mouse substantia nigra. Exp. Neurol. 176, 322–327 10.1006/exnr.2002.7946 12359173

[B32] Hoarau J. J. Krejbich-Trotot P. Jaffar-Bandjee M. C. Das T. Thon-Hon G. V. Kumar S. (2011). Activation and control of CNS innate immune responses in health and diseases: a balancing act finely tuned by Neuroimmune Regulators (NIReg). CNS Neurol. Disord. Drug Targets 10, 25–43 10.2174/187152711794488601 21143144

[B33] Hollingworth P. Harold D. Sims R. Gerrish A. Lambert J.-C. Carrasquillo M. M. (2011). Common variants at ABCA7, MS4A6A/MS4A4E, EPHA1, CD33 and CD2AP are associated with Alzheimer's disease. Nat. Genet. 43, 429–435 10.1038/ng.803 21460840 PMC3084173

[B34] Holmes C. Cunningham C. Zotova E. Culliford D. Perry V. H. (2011). Proinflammatory cytokines, sickness behavior, and Alzheimer disease. Neurology 77, 212–218 10.1212/WNL.0b013e318225ae07 21753171 PMC3136056

[B35] Hulse R. E. Swenson W. G. Kunkler P. E. White D. M. Kraig R. P. (2008). Monomeric IgG is neuroprotective via enhancing microglial recycling endocytosis and TNF-alpha. J. Neurosci. 28, 12199–12211 10.1523/JNEUROSCI.3856-08.2008 19020014 PMC2699401

[B36] Kam T.-I. Song S. Gwon Y. Park H. Yan J.-J. Im I. (2013). FcγRIIb mediates amyloid-β neurotoxicity and memory impairment in Alzheimer's disease. J. Clin. Invest. 123, 2791–2802 10.1172/JCI66827 23921129 PMC3696552

[B37] Katsuki H. Nakai S. Hirai Y. Akaji K. Kiso Y. Satoh M. (1990). Interleukin-1-Beta inhibits long-term potentiation in the CA3 region of mouse hippocampal slices. Eur. J. Pharmacol. 181, 323–326 10.1016/0014-2999(90)90099-R 2166677

[B38] Komine-Kobayashi M. Chou N. Mochizuki H. Nakao A. Mizuno Y. Urabe T. (2004). Dual role of Fcγ receptor in transient focal cerebral ischemia in mice. Stroke 35, 958–963 10.1161/01.STR.0000120321.30916.8E 14988576

[B39] Lambert J. C. Heath S. Even G. Campion D. Sleegers K. Hiltunen M. (2009). Genome-wide association study identifies variants at CLU and CR1 associated with Alzheimer's disease. Nat. Genet. 41, 1094–1099 10.1038/ng.439 19734903

[B40] Lambert J.-C. Ibrahim-Verbaas C. A. Harold D. Naj A. C. Sims R. Bellenguez C. (2013). Meta-analysis of 74,046 individuals identifies 11 new susceptibility loci for Alzheimer's disease. Nat. Genet. 45, 1452–U206 10.1038/ng.2802 24162737 PMC3896259

[B41] Le W. D. Rowe D. Xie W. J. Ortiz I. He Y. Appel S. H. (2001). Microglial activation and dopaminergic cell injury: an *in vitro* model relevant to Parkinson's disease. J. Neurosci. 21, 8447–8455 11606633 10.1523/JNEUROSCI.21-21-08447.2001 PMC6762816

[B42] Lemere C. A. Masliah E. (2010). Can Alzheimer disease be prevented by amyloid-beta immunotherapy? Nat. Rev. Neurol. 6, 108 10.1038/nrneurol.2009.219 20140000 PMC2864089

[B43] Li Y. N. Qin X. J. Kuang F. Wu R. Duan M. L. Ju G. (2008). Alterations of Fc gamma receptor I and Toll-like receptor 4 mediate the antiinflammatory actions of microglia and astrocytes after adrenaline-induced blood-brain barrier opening in rats. J. Neurosci. Res. 86, 3556–3565 10.1002/jnr.21810 18756515

[B44] Lu J. Moochhala S. Kaur C. Ling E. A. (2001). Cellular inflammatory response associated with breakdown of the blood-brain barrier after closed head injury in rats. J. Neurotrauma 18, 399–408 10.1089/089771501750170976 11336441

[B45] Lunnon K. Teeling J. L. Tutt A. L. Cragg M. S. Glennie M. J. Perry V. H. (2011). Systemic inflammation modulates Fc receptor expression on microglia during chronic neurodegeneration. J. Immunol. 186, 7215–7224 10.4049/jimmunol.0903833 21572034

[B46] Mantovani A. Sica A. Sozzani S. Allavena P. Vecchi A. Locati M. (2004). The chemokine system in diverse forms of macrophage activation and polarization. Trends Immunol. 25, 677–686 10.1016/j.it.2004.09.015 15530839

[B47] Masters C. L. Simms G. Weinman N. A. Multhaup G. McDonald B. L. Beyreuther K. (1985). Amyloid plaque core protein in alzheimer-disease and down syndrome. Proc. Natl. Acad. Sci. U.S.A. 82, 4245–4249 10.1073/pnas.82.12.4245 3159021 PMC397973

[B48] Mohamed H. A. Mosier D. R. Zou L. L. Siklos L. Alexianu M. E. Engelhardt J. I. (2002). Immunoglobulin Fc gamma receptor promotes immunoglobulin uptake, immunoglobulin-mediated calcium increase, and neurotransmitter release in motor neurons. J. Neurosci. Res. 69, 110–116 10.1002/jnr.10271 12111822

[B49] Morgan D. (2009). The role of microglia in antibody-mediated clearance of amyloid-Beta from the brain. CNS Neurol. Disord. Drug Targets 8, 7–15 10.2174/187152709787601821 19275633

[B50] Mosser D. M. (2003). The many faces of macrophage activation. J. Leukoc. Biol. 73, 209–212 10.1189/jlb.0602325 12554797

[B51] Mosser D. M. Edwards J. P. (2008). Exploring the full spectrum of macrophage activation. Nat. Rev. Immunol. 8, 958–969 10.1038/nri2448 19029990 PMC2724991

[B52] Mullan M. Crawford F. Axelman K. Houlden H. Lilius L. Winblad B. (1992). A pathogenic mutation for probable Alzheimer's disease in the APP gene at the N terminus of beta amyloid. Nat. Genet. 1, 345–347 10.1038/ng0892-345 1302033

[B53] Murinello S. Mullins R. F. Lotery A. J. Perry V. H. Teeling J. L. (2014). Fc gamma receptor upregulation is associated with immune complex inflammation in the mouse retina and early age-related macular degeneration. Invest. Ophthalmol. Vis. Sci. 55, 247–258 10.1167/iovs.13-11821 24334446 PMC3891269

[B54] Nakahara J. Tan-Takeuchi K. Seiwa C. Gotoh M. Kaifu T. Ujike A. (2003). Signaling via immunoglobulin Fc receptors induces oligodendrocyte precursor cell differentiation. Dev. Cell 4, 841–852 10.1016/S1534-5807(03)00155-2 12791269

[B55] Nakamura K. Hirai H. Torashima T. Miyazaki T. Tsurui H. Xiu Y. (2007). CD3 and immunoglobulin G Fc receptor regulate cerebellar functions. Mol. Cell. Biol. 27, 5128–5134 10.1128/MCB.01072-06 17502348 PMC1951947

[B56] Nimmerjahn F. Ravetch J. V. (2008). Fcgamma receptors as regulators of immune responses. Nat. Rev. Immunol. 8, 34–47 10.1038/nri2206 18064051

[B57] Nitsch R. M. Hock C. (2008). Targeting beta-amyloid pathology in Alzheimer's disease with A beta immunotherapy. Neurotherapeutics 5, 415–420 10.1016/j.nurt.2008.05.013 18625453 PMC5084243

[B58] Nyland H. Mork S. Matre R. (1984). Fc gamma receptors in multiple sclerosis brains. Ann. N.Y. Acad. Sci. 436, 476–479 10.1111/j.1749-6632.1984.tb14823.x

[B59] Olson M. I. Shaw C.-M. (1969). Presenile dementia and Alzheimer disease in mongolism. Brain 92, 147–156 10.1093/brain/92.1.147 4237656

[B60] Orr C. F. Rowe D. B. Mizuno Y. Mori H. Halliday G. M. (2005). A possible role for humoral immunity in the pathogenesis of Parkinson's disease. Brain 128, 2665–2674 10.1093/brain/awh625 16219675

[B61] Peress N. S. Fleit H. B. Perillo E. Kuljis R. Pezzullo C. (1993). Identifcation of Fc gamma RI, II and III on normal human brain ramified microglia in senile plaques in Alzheimer's disease. J. Neuroimmunol. 48, 71–80 10.1016/0165-5728(93)90060-C 8227309

[B62] Poduslo J. F. Curran G. L. Berg C. T. (1994). Macromolecular permeability across the blood-nerve and blood-brain barriers. Proc. Natl. Acad. Sci. U.S.A. 91, 5705–5709 10.1073/pnas.91.12.5705 8202551 PMC44065

[B63] Puentener U. Booth S. G. Perry V. H. Teeling J. L. (2012). Long-term impact of systemic bacterial infection on the cerebral vasculature and microglia. J. Neuroinflammation 9:146 10.1186/1742-2094-9-146 22738332 PMC3439352

[B64] Reisberg B. Doody R. Stoffler A. Schmitt F. Ferris S. Mobius H. J. (2003). Memantine in moderate-to-severe Alzheimer's disease. N. Engl. J. Med. 348, 1333–1341 10.1056/NEJMoa013128 12672860

[B65] Rinne J. O. Brooks D. J. Rossor M. N. Fox N. C. Bullock R. Klunk W. E. (2010). (11)C-PiB PET assessment of change in fibrillar amyloid-beta load in patients with Alzheimer's disease treated with bapineuzumab: a phase 2, double-blind, placebo-controlled, ascending-dose study. Lancet Neurol. 9, 363–372 10.1016/S1474-4422(10)70043-0 20189881

[B66] Ross F. M. Allan S. M. Rothwell N. J. Verkhratsky A. (2003). A dual role for interleukin-1 in LTP in mouse hippocampal slices. J. Neuroimmunol. 144, 61–67 10.1016/j.jneuroim.2003.08.030 14597099

[B67] Salloway S. Sperling R. Fox N. C. Blennow K. Klunk W. Raskind M. (2014). Two phase 3 trials of bapineuzumab in mild-to-moderate Alzheimer's disease. N. Engl. J. Med. 370, 322–333 10.1056/NEJMoa1304839 24450891 PMC4159618

[B68] Salloway S. P. Black R. Sperling R. Fox N. Gilman S. Schenk D. (2010). A phase 2 multiple ascending dose trial of bapineuzumab in mild to moderate Alzheimer disease reply. Neurology 74, 2026–2027 10.1212/WNL.0b013e3181e03844 19923550 PMC2790221

[B69] Schenk D. (2002). Amyloid-beta immunotherapy for Alzheimer's disease: the end of the beginning. Nat. Rev. Neurosci. 3, 824–828 10.1038/nrn938 12360327

[B70] Schlachetzki F. Zhu C. N. Pardridge W. M. (2002). Expression of the neonatal Fc receptor (FcRn) at the blood-brain barrier. J. Neurochem. 81, 203–206 10.1046/j.1471-4159.2002.00840.x 12067234

[B71] Stellwagen D. Malenka R. C. (2006). Synaptic scaling mediated by glial TNF-alpha. Nature 440, 1054–1059 10.1038/nature04671 16547515

[B72] Strittmatter W. J. Roses A. D. (1995). Apolipoprotein E and Alzheimer's disease. Proc. Natl. Acad. Sci. U.S.A. 92, 4725–4727 10.1073/pnas.92.11.4725 7761390 PMC41779

[B73] Takai T. Li M. Sylvestre D. Clynes R. Ravetch J. V. (1994). FcR gamma-chain deletion results in pleiotropic effector cell defects. Cell 76, 519–529 10.1016/0092-8674(94)90115-5 8313472

[B74] Tariot P. N. Farlow M. R. Grossberg G. T. Graham S. M. McDonald S. Gergel I. (2004). Memantine treatment in patients with moderate to severe Alzheimer disease already receiving donepezil: a randomized controlled trial. JAMA 291, 317–324 10.1001/jama.291.3.317 14734594

[B75] Teeling J. L. Carare R. O. Glennie M. J. Perry V. H. (2012). Intracerebral immune complex formation induces inflammation in the brain that depends on Fc receptor interaction. Acta Neuropathol. 124, 479–490 10.1007/s00401-012-0995-3 22618994 PMC3444701

[B76] Ulvestad E. Williams K. Vedeler C. Antel J. Nyland H. Mork S. (1994). Reactive microglia in multiple sclerosis lesions have an increased expression of receptors for the Fc part of IgG. J. Neurol. Sci. 121, 125–131 10.1016/0022-510X(94)90340-9 8158203

[B77] Wilcock D. M. Alamed J. Gottschall P. E. Grimm J. Rosenthal A. Pons J. (2006). Deglycosylated anti-amyloid-beta antibodies eliminate cognitive deficits and reduce parenchymal amyloid with minimal vascular consequences in aged amyloid precursor protein transgenic mice. J. Neurosci. 26, 5340–5346 10.1523/JNEUROSCI.0695-06.2006 16707786 PMC6675288

[B78] Wilcock D. M. Munireddy S. K. Rosenthal A. Ugen K. E. Gordon M. N. Morgan D. (2004a). Microglial activation facilitates Abeta plaque removal following intracranial anti-Abeta antibody administration. Neurobiol. Dis. 15, 11–20 10.1016/j.nbd.2003.09.015 14751766

[B79] Wilcock D. M. Rojiani A. Rosenthal A. Levkowitz G. Subbarao S. Alamed J. (2004b). Passive amyloid immunotherapy clears amyloid and transiently activates microglia in a transgenic mouse model of amyloid deposition. J. Neurosci. 24, 6144–6151 10.1523/JNEUROSCI.1090-04.2004 15240806 PMC6729674

[B80] Wilcock D. M. Rojiani A. Rosenthal A. Subbarao S. Freeman M. J. Gordon M. N. (2004c). Passive immunotherapy against Abeta in aged APP-transgenic mice reverses cognitive deficits and depletes parenchymal amyloid deposits in spite of increased vascular amyloid and microhemorrhage. J. Neuroinflammation 1, 24 10.1186/1742-2094-1-24 15588287 PMC539292

[B81] Wilcock D. M. Zhao Q. Morgan D. Gordon M. N. Everhart A. Wilson J. G. (2011). Diverse inflammatory responses in transgenic mouse models of Alzheimer's disease and the effect of immunotherapy on these responses. ASN Neuro 3, 249–258 10.1042/AN20110018 21995345 PMC3227004

[B82] Wyss-Coray T. Lin C. Yan F. R. Yu G. Q. Rohde M. McConlogue L. (2001). TGF-beta 1 promotes microglial amyloid-beta clearance and reduces plaque burden in transgenic mice. Nat. Med. 7, 612–618 10.1038/87945 11329064

[B83] Yang L. Lindholm K. Konishi Y. Li R. Shen Y. (2002). Target depletion of distinct tumor necrosis factor receptor subtypes reveals hippocampal neuron death and survival through different signal transduction pathways. J. Neurosci. 22, 3025–3032 11943805 10.1523/JNEUROSCI.22-08-03025.2002 PMC6757531

[B84] Yuasa T. Kubo S. Yoshino T. Ujike A. Matsumura K. Ono M. (1999). Deletion of Fc gamma receptor IIB renders H-2(b) mice susceptible to collagen-induced arthritis. J. Exp. Med. 189, 187–194 10.1084/jem.189.1.187 9874575 PMC1887699

[B85] Zhang Y. Pardridge W. M. (2001). Mediated efflux of IgG molecules from brain to blood across the blood-brain barrier. J. Neuroimmunol. 114, 168–172 10.1016/S0165-5728(01)00242-9 11240028

[B86] Zotova E. Bharambe V. Cheaveau M. Morgan W. Holmes C. Harris S. (2013). Inflammatory components in human Alzheimer's disease and after active amyloid-beta(42) immunization. Brain 136, 2677–2696 10.1093/brain/awt210 23943781

